# Environmental and Physiological Influences to Isotopic Ratios of N and Protein Status in a Montane Ungulate in Winter

**DOI:** 10.1371/journal.pone.0103471

**Published:** 2014-08-07

**Authors:** David D. Gustine, Perry S. Barboza, Layne G. Adams, Nathan B. Wolf

**Affiliations:** 1 United States Geological Survey, Alaska Science Center, Anchorage, Alaska, United States of America; 2 Institute of Arctic Biology, Department of Biology and Wildlife, University of Alaska Fairbanks, Fairbanks, Alaska, United States of America; 3 Environment and Natural Resources Institute, University of Alaska Anchorage, Anchorage, Alaska, United States of America; Institut Pluridisciplinaire Hubert Curien, France

## Abstract

Winter severity can influence large herbivore populations through a reduction in maternal proteins available for reproduction. Nitrogen (N) isotopes in blood fractions can be used to track the use of body proteins in northern and montane ungulates. We studied 113 adult female caribou for 13 years throughout a series of severe winters that reduced population size and offspring mass. After these severe winters, offspring mass increased but the size of the population remained low. We devised a conceptual model for routing of isotopic N in blood in the context of the severe environmental conditions experienced by this population. We measured δ^15^N in three blood fractions and predicted the relative mobilization of dietary and body proteins. The δ^ 15^N of the body protein pool varied by 4‰ and 46% of the variance was associated with year. Annual variation in δ^15^N of body protein likely reflected the fall/early winter diet and winter locations, yet 15% of the isotopic variation in amino acid N was due to body proteins. Consistent isotopic differences among blood N pools indicated that animals tolerated fluxes in diet and body stores. Conservation of body protein in caribou is the result of active exchange among diet and body N pools. Adult females were robust to historically severe winter conditions and prioritized body condition and survival over early investment in offspring. For a vagile ungulate residing at low densities in a predator-rich environment, protein restrictions in winter may not be the primary limiting factor for reproduction.

## Introduction

Resource availability for large herbivores is primarily dictated by environmental conditions and intraspecific competition [1]. In northern and montane systems, short growing seasons (∼2–3 months) are followed by extended periods of resource scarcity throughout winter. Winter severity is primarily associated with snowfall as deep snowpacks reduce food availability, increase the costs of acquiring foods, and reduce the length of the forthcoming growing season [2], [3]. Severe winters can influence population trajectories, in part, through a reduction in maternal resources available for reproduction and the subsequent growth and survival of offspring [4]–[7]. Nutrient allocation strategies of females throughout winter must therefore balance the tradeoff of accruing and maintaining sufficient resources to survive uncertain conditions and demands of pregnancy and lactation. For animals adapted to harsh and variable conditions of northern and alpine environments, plasticity in allocation [8] and timing of reproduction [9] temper the effect of large fluctuations in resource availability.

Nutrient allocation in reproductive females occurs along a continuum of capital to income investment [10], [11]. Nutrient partitioning to offspring may shift in response to changes in availability of forages, availability of maternal nutrients, and the nutrient demands of gestation and lactation [12], [13]. Thus, allocation of capital and income resources for a reproductive female is a function of the availability and demand for any particular nutrient. Additionally, demand may be altered through environmental constraint or maternal restraint [14]. When resources are limited, females may reduce their own reproductive costs by reducing their investment in fetal growth and milk production [15], [16]. This conservation of endogenous resources to enhance maternal survival and increase the likelihood of future breeding opportunities has been termed the “selfish” female hypothesis (e.g., *Rangifer tarandus* [13] and *Ovis canadensis* [17]). The controls on accruing, maintaining, and mobilizing nutrient stores for reproduction can vary substantially within and among species by body size, food availability, offspring mass, and milk production. Some northern and alpine ungulates, such as muskoxen (*Ovibos moschatus*) [18], mountain goats (*Oreamnos americanus*) [19], [20], and bighorn sheep (*Ovis canadensis*) [17] rely solely on stored capital for production of young. Alternatively, others, such as caribou (*R. tarandus*) use primarily capital and some income to meet nutrient demands throughout a reproductive event [21], [22], whereas roe deer (*Capreolus capreolus*) rely exclusively on income to produce offspring [12].

Availability of maternal proteins for reproduction may provide the mechanistic link between environmental conditions, investment in offspring, and population productivity [23], [24]. Foraging constraints and high energy demands during severe winters may deplete protein stores that are needed for reproduction [25]. Demands for energy and protein increase progressively through late winter into spring as pregnancy ends and milk production increases in the first two to three weeks after parturition [26]. Protein demands for reproductive females peak during lactation, which typically occurs within the first few weeks of the growing season [27]. Nutrient requirements for lactation commonly necessitate the use of maternal capital as well as the increased intake of forages [12], [19]. Winters with deep snow affect maternal investment of caribou by reducing birth mass of offspring, which may decrease calf survival and recruitment [7]. Additionally, in preparation for the subsequent breeding season, depleted protein reserves must be replenished as the growing season progresses and lactation demands decrease. Therefore, the ability of reproductive females to maintain and allocate stores of maternal proteins throughout variable and often severe environmental conditions for late gestation and lactation is crucial for the production, growth, and survival of offspring [21], [28].

**Figure 1:**
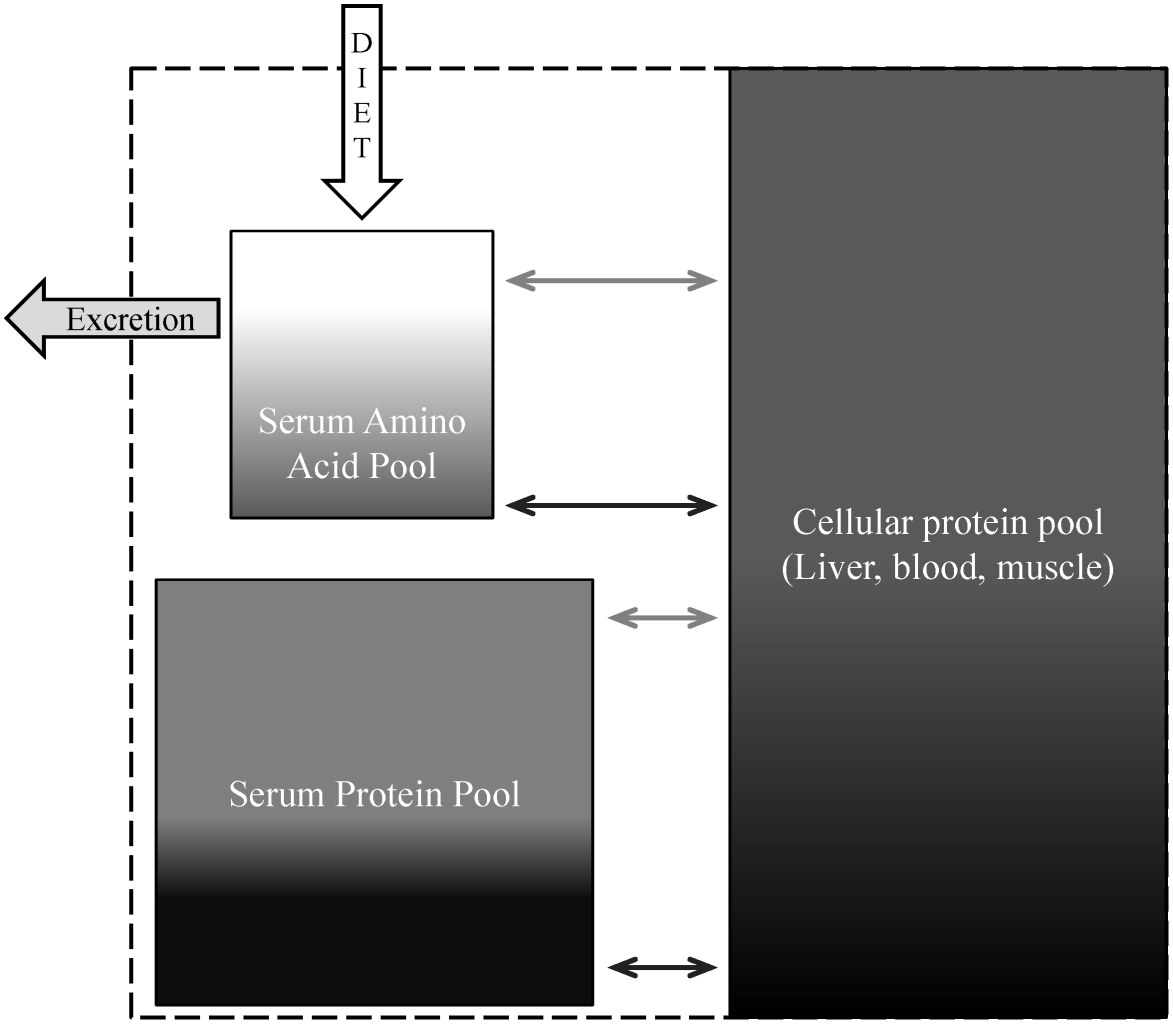
A conceptual model of the routing of isotopes of N within a northern ungulate during winter. The size of each box indicates the relative size of each pool of N. The gradient of shading in each box indicates the range from less to more ^15^N. Lighter arrows indicate flows of depleted N when animals are in positive N balance and gaining lean mass, while darker arrows indicate flows of enriched N when animals are losing lean mass.

**Figure 2:**
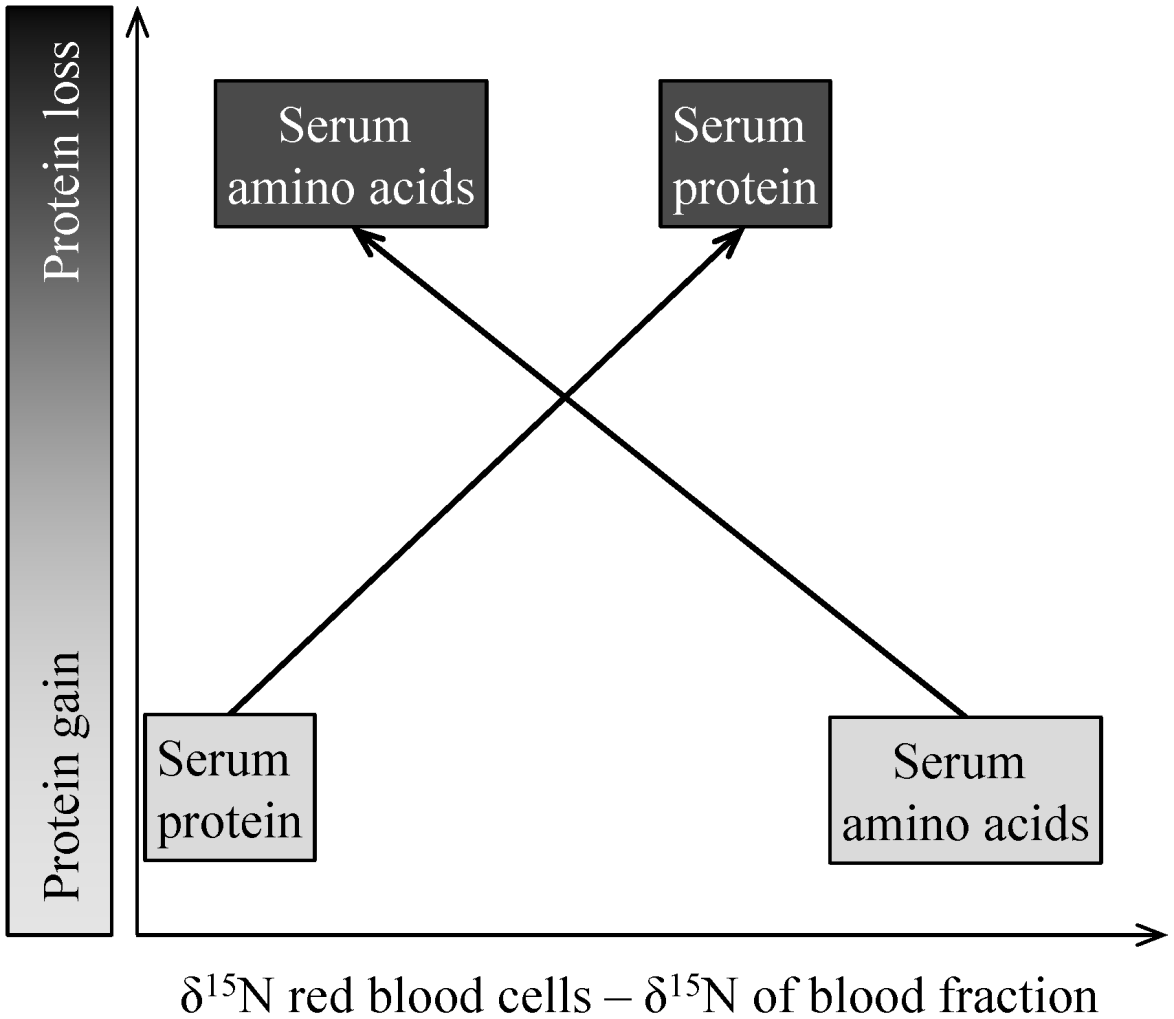
Projected differences in isotopic ratios of N (δ^15^N in ‰) between red blood cells and serum fractions in relation to gain or loss of body protein; the gradient of shading indicates the light (low δ^15^N) to heavy N (high δ^15^N).

Isotopes of nitrogen (N) can be used to assess the mobilization of body proteins in northern and montane ungulates because the isotope ratio of heavy ^15^N to light ^14^N (δ^15^N) is typically higher in body proteins than in dietary proteins [29]. Isotopically light N is preferentially incorporated in metabolic processes and excreted, thereby leaving body tissues with more of the heavy isotope [30]. Nitrogen in dietary proteins is digested and absorbed as amino acids and those amino acids can be incorporated into body tissues as protein. If the dietary amino acids are used for energy, glucogenesis, or lipogenesis, the N is removed and excreted as urea (Fig. 1) [31]. Thus, increasing δ^15^N ratios in serum amino acids indicate that an increasing proportion of the associated N is derived from body proteins [18]. As amino acids are reused in the body, the δ^15^N of serum proteins rises above cellular proteins (e.g., red blood cells), so δ^15^N in serum protein provides an additional indicator of N conservation in wintering ungulates (Fig. 2). While δ^15^N of bulk serum samples may occasionally correspond to protein status [29], [32], bulk isotopic values obscure the complexities of N dynamics within pools of N in the blood of wintering ungulates. Therefore, δ^15^N of blood fractions (i.e., red blood cells, serum proteins, and serum amino acids) and relative changes therein (Fig. 1, 2) provide a conceptual blood-based system for evaluating protein status and allocation of maternal proteins in wintering ungulates [33].

Several environmental and physiological factors act in concert to shape the isotopic composition of animals [34]. In addition to protein status, factors such as age and body mass may alter isotopic incorporation rates and hence the isotopic composition of tissues [35]. Caribou, as in other migratory species [36], integrate isotopic “signals” across diverse environmental and isotopic gradients (i.e., isoscapes) [37] these isotopic gradients should be detectable within components of their tissues.

**Figure 3:**
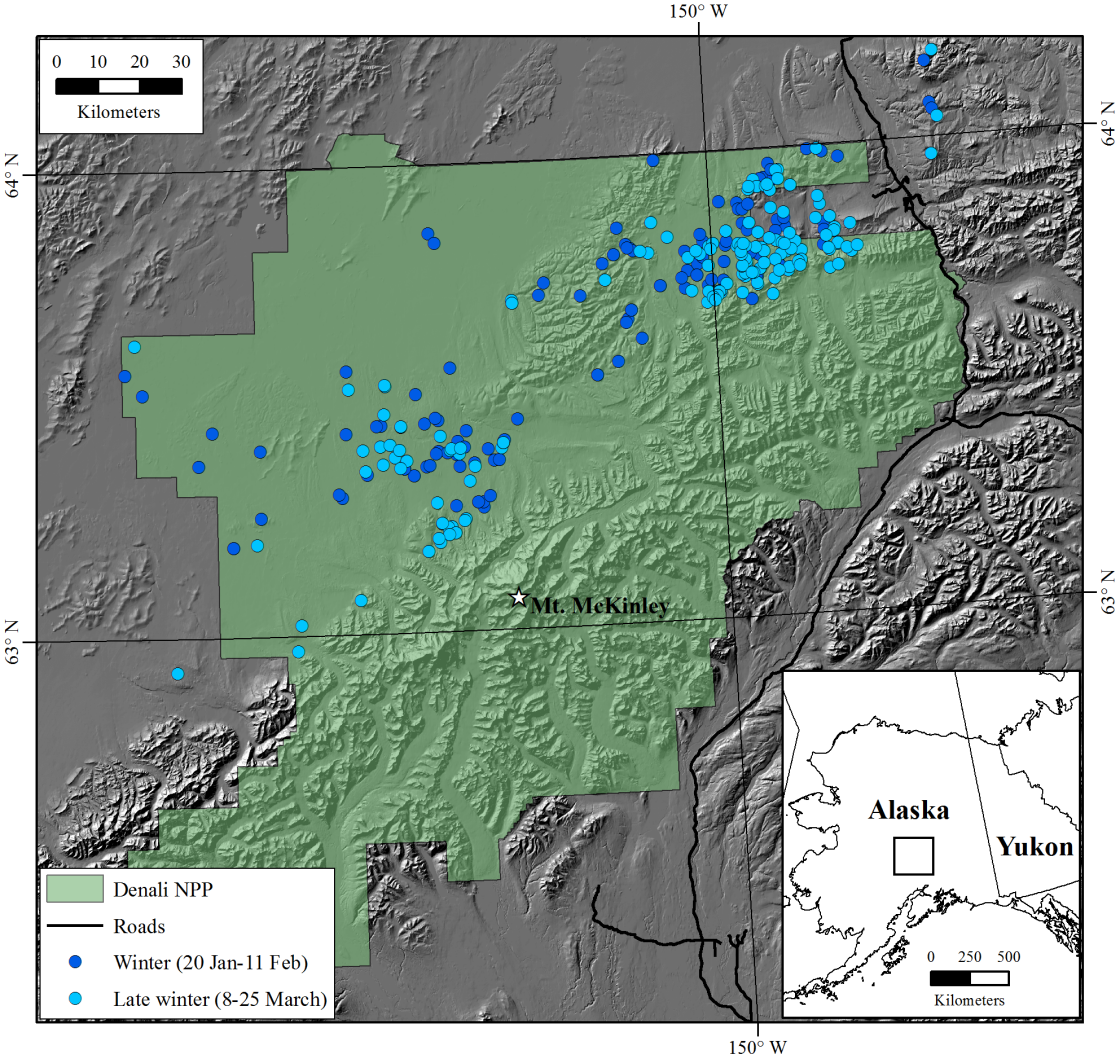
Winter and late winter locations of adult female caribou in Denali National Park and Preserve (Denali NPP), Alaska; blood was collected (*n* = 168) for isotopic analyses at late winter locations during March 1993–2007.

**Figure 4:**
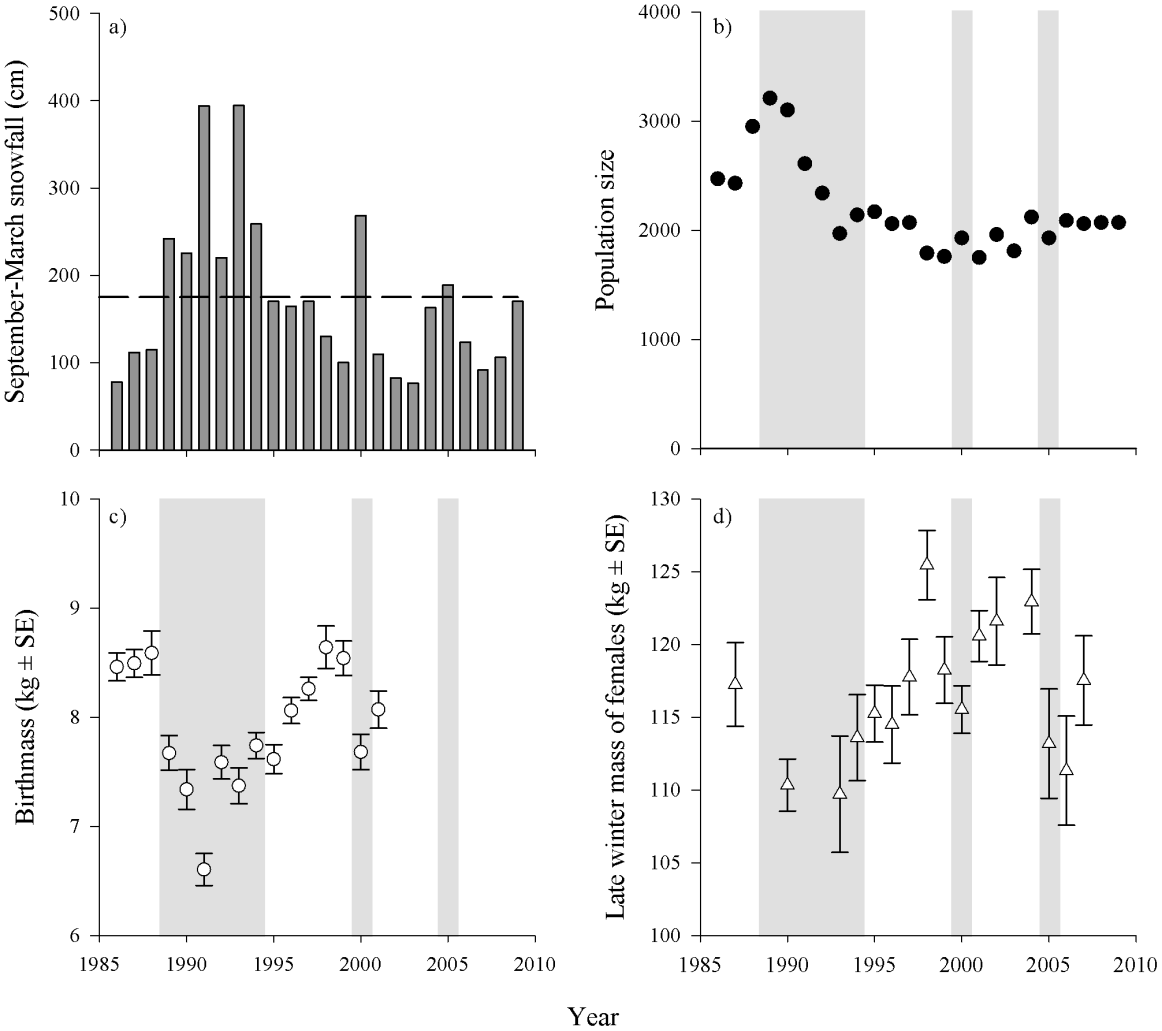
The a) snowfall (22-year mean depicted by dashed line), b) size of the Denali Caribou Herd, 1986–2009 [40], c) birth masses of neonates [7], and d) body masses of adult females (≥3 y) in late winter (L. Adams, USGS, unpublished data). Shading denotes winters with above average snowfall.

We used a conceptual model of isotopic routing of N (Fig. 1) to assess potential correlates to characteristics of isotopic N and to evaluate protein dynamics in adult female caribou of the Denali herd through the winters of 1993–2002, 2004–2005, and 2007. The Denali herd is a low density (≤0.3 caribou km^−2^) population that resides in a multi-predator multi-prey system within Denali National Park and Preserve (Fig. 3) that is largely insulated from anthropogenic influences [38]. Adult females are large bodied (Fig. 4d) and experience high neonatal losses to predation [38], [39]. The Denali herd increased from 2,500 to 3,200 in 1989 and then declined abruptly, to approximately 2,000 caribou, during 6 consecutive years of above-average winter snowfall (1988–1994) with the winter of 1992–93 the harshest winter on record (Fig. 4a, b) [40]. The period of severe winter conditions resulted in increased calf and adult mortality [38]–[40]; reduced birth masses of calves (Fig. 4c) [7]; and reduced lactational investment in offspring [6]. We used blood samples and female characteristics (age, mass, and winter location) in late winter to evaluate the long-term patterns and sources of variation in δ^15^N. We employed our conceptual model of isotopic N in blood fractions from late-winter collections to assess protein status of females through a wide range of winter severity. We predicted that the δ^15^N of serum proteins and amino acids would enrich with snowfall, thus, the δ^15^N of serum proteins would rise above the δ^15^N of red blood cells while δ^15^N of serum amino acids would approach the δ^15^N of red blood cells.

## Materials and Methods

### Ethics Statement

Capture and handling of animals reported herein comply with scientific guidelines and permits acquired from the State of Alaska and Denali National Park and Preserve, National Park Service, Healy, Alaska, USA.

### Study area

The annual range (16,000 km^2^) of the Denali herd included most of Denali National Park and Preserve, which was roughly bisected by the Alaska Range (Fig. 3). Climate in the region is subarctic-montane: average annual temperatures were below freezing (−3°C), with most annual precipitation (average = 38 cm) falling as snow between September and May. Caribou wintered in the northern part of Denali National Park and Preserve (L. Adams, U.S. Geological Survey, unpublished data), which was typified by boreal-tussock lowlands separated by mountains (approx. 600–1,300 m). Vegetation types below the 800 m treeline consisted of sedge (*Carex* spp.)-shrub (*Salix* spp. and *Betula* spp.), tussock tundra, and spruce (*Picea* spp.) and deciduous (*Populus* spp. and *Betula papyrifera*) woodlands, whereas alpine areas contained a mixture of talus, scree, sedge, mountain avens (*Dryas* spp.), heath (*Cassiope* spp.), graminoids, lichens, and mosses.

### Collection and processing of blood samples

Since 1986, research on the Denali herd has relied on capturing female caribou via helicopter darting; radiocollaring, weighing, and blood-sampling those individuals; and periodically monitoring collared individuals throughout the year [41]. The ages of captured individuals were either known (i.e., captured as calves) or estimated from cementum annuli of teeth extracted during the initial capture [42]. Reproductive status was confirmed by presence of a calf or antlers and (or) udder status [43]. Results reported here are based on late-winter (8–25 Mar) captures and blood samples and mid- (20 Jan–11 Feb) and late-winter radiolocations of adult (≥3 y) female caribou from 1993 to 2007 (Fig. 3).

During captures, blood (30 mL) was collected from either the saphenous or cephalic veins with a 30 ml syringe and a 20 gauge×1.0 inch needle. Whole blood was immediately transferred into 10 mL serum collection tubes and allowed to clot in the tubes before separating serum. In the field, samples were kept from freezing in the helicopter until processing. Blood samples were centrifuged at 3,000×*g* at room temperature for 15–20 minutes. Serum was pipetted into 1.8 mL cryovials and decanted to leave a drained blood clot (red blood cells) in the collection tube; whole serum and blood clots were stored at −20°C until processing.

We used sodium tungstate (Na_2_WO_4_) to precipitate serum proteins from whole serum. Whole serum (1.5 mL) was added to a thoroughly mixed solution of 0.66 NH_2_SO_4_ (1.5 mL at 3.23 mL·100 ml^−1^), water (3 mL), and Na_2_WO_4_ (1.5 mL at 10% w:v) at room temperature. Denatured serum proteins were precipitated by centrifugation at 10,000×*g* for 20 minutes at 10°C [44]. The supernatant was decanted and retained for isolation of amino acids. The protein pellet was re-suspended in distilled water (8 mL) and centrifuged again to remove residual solutes from the protein precipitate. Serum amino acids were isolated and stored as in Gustine et al. [18]. Serum proteins and amino acid elutions were lyophilized for analysis (Freeze Dryer 8, Labconco, Kansas City, MO).

Isotope ratio mass spectrometry was used to measure ^15^N/^14^N in blood fractions (red blood cells, and serum proteins and amino acids) against atmospheric N (δ in ‰) [45] at the Alaska Stable Isotope Facility and Forest Soils Laboratory at the University of Alaska Fairbanks. This system was accurate within 0.39‰ of peptone from meat. We assumed the following: 1) δ^15^N of red blood cells (δ^15^N_RBC_) largely represented body proteins that were deposited <90 d prior to collection [46]; 2) the δ^15^N of serum proteins (δ^15^N_Proteins_) represented the pool of albumins and globulins that interchanged primarily with cellular proteins [47] and were likely deposited within the previous 4–12 d [48]; and the δ^15^N of serum amino acids represented recent (<4 d) modifications of dietary and (or) endogenous sources of N [49], [50]. From the conceptual model of isotopic routing (Fig. 1), we calculated two indices of protein status derived from N fractions in the blood of each caribou: the difference between δ^15^N_RBC_ and δ^15^N_Proteins_ (Δ_RBC-Proteins_) and the difference between δ^15^N_RBC_ and δ^15^N_AAs_ (Δ_RBC-AAs_). We expected that the Δ_RBC-Proteins_ would increase as maternal protein was mobilized, as recycling of body N results in an increase in δ^15^N of serum proteins. Secondly, we expected the Δ_RBC-AAs_ would decrease with losses of maternal protein as the pool of amino acids became increasingly derived from endogenous sources of N (Fig. 2).

### Statistical analyses

We used descriptive statistics to report age, body mass, and isotopic parameters (δ^15^N_RBC_, δ^15^N_Proteins_, δ^15^N_AAs_, Δ_RBC-Proteins_, and Δ_RBC-AAs_) by year. We used multiple regression [51] to examine the relative importance and effects of year, age, body mass, mid-winter or late winter locations, and δ^15^N_RBC_ on isotopic parameters (Table 1). Only individuals with data for all variables for a given isotopic parameter were included in a model. Conservative tolerance scores (<0.40) were used to evaluate multi-collinearity among the set of independent variables. Age (y) and body mass (kg) were measured at the time blood was sampled. Age was entered as a quadratic term to account for any effect of maturation and senescence on isotopic composition and protein status. Spatial locations were the Universal Transverse Mercator easting and northing coordinates of relocations of each animal prior to (mid-winter) or at capture (late winter). To reflect the substantial differences in turnover times of each isotope within caribou, temporally appropriate spatial locations were used as covariates within models for each isotope and metric of protein status: mid-winter for δ^15^N_RBC_, δ^15^N_Proteins_, and Δ_RBC-Proteins_ and late winter for δ^15^N_AAs_ and Δ_RBC-AAs_. The adjusted coefficient of determination (adjusted *R* ^2^) was used to estimate the independent effect of each covariate to each isotopic parameter [52]. The change in adjusted *R* ^2^ after the covariate was removed from the full model (ΔAdj. *R* ^2^ *_i_*) was used as an index of the relative importance of covariate*_i_*, which was interpreted as the percent of variance explained by covariate*_i_* within the model. We used coefficients (β), robust estimates of variance [53], and 95% confidence intervals to evaluate parameters in each model.

**Table 1:** Models to examine the potential sources of variation in the isotopic ratios of nitrogen (δ^15^N) in red blood cells (δ^15^N_RBC_), serum proteins (δ^15^N_Proteins_), and serum amino acids (δ^15^N_AAs_) as well as the differences between δ^15^N_RBC_ and δ^15^N_Proteins_ (Δ_RBC-proteins_) and δ^15^N_RBC_ and δ^15^N_AAs_ (Δ_RBC-AAs_) in adult (≥3 y) female caribou in Denali National Park and Preserve, Alaska, March, 1993–2007.

Dependent variable	Model	*n*	*df*	*F*	Adjusted *R* ^2^
δ^15^N_RBC_	Year+age+age^2^+ body mass+winter location^a^	116	16	12.74	0.62
δ^15^N_Proteins_	Year+age+age^2^+ body mass+winter location^a^+δ^15^N_RBC_	91	16	21.28	0.78
δ^15^N_AAs_	Year+age+age^2^+ body mass+late winter location^b^+δ^15^N_RBC_	118	18	3.95	0.31
Δ_RBC-proteins_	Year+age+age^2^+ body mass+winter location^a^	91	15	10.69	0.62
Δ_RBC-AAs_	Year+age+age^2^+ body mass+late winter location^b^	118	17	3.84	0.29

a Winter locations were acquired 20 Jan–11 Feb, 1–2 months prior to blood sampling.

b Late winter locations were acquired 8–25 Mar at blood sampling.

We used linear regression to examine the relationships between snowfall (cm) and each isotopic parameter in the blood. Snowfall (Fig. 1a) was measured from September 1-March 30. We assumed the variance accounted for by snowfall would lie within the year effect, thus we regressed the β of year for each isotopic parameter from the above multiple regression models on snowfall. We predicted positive slopes for δ^15^N_RBC_, δ^15^N_Proteins_, δ^15^N_AAs_, and Δ_RBC-Proteins_ and a negative slope for Δ_RBC-AAs_. Coefficients of determination (*r* ^2^) and 95% confidence intervals were used to evaluate strength and direction (β≠0) of the relationships, respectively. A Shapiro-Wilk’s test was used to evaluate the assumption of normality for all comparisons [51]. Unless specified otherwise, results are reported as means (*x*-) ± SD.

## Results

We collected 168 blood samples from 113 (pregnancy rate = 95%) caribou during 13 of 15 years during our study (Table 2). We treated samples collected from an individual in different years (*n* = 65) as independent because these samples were generally collected 3–4 years apart. Some samples of blood fractions were lost during handling, extraction, or counting of isotopes (8%, 17%, and 19% for δ^15^N_RBC_, δ^15^N_Proteins_, and δ^15^N_AAs_, respectively) subsequently reducing sample sizes for Δ_RBC-Proteins_ and Δ_RBC-AAs_ by 25% (Table 3).

**Table 2:** Ages, body masses, and number of winter (20 January to 11 February) and late winter (8–25 March) locations of radiocollared adult (≥3 y) female caribou with corresponding blood samples in Denali National Park and Preserve, Alaska.

Year	Age (y)			Body mass (kg)			Locations	
	*n*	*x*-	SD	*n*	*x*-	SD	Winter	Late winter
1993	14	5.7	2.2	13	111.0	14.7	11	13
1994	9	3.7	1.3	9	112.4	9.1	9	9
1995	13	6.2	0.6	12	115.3	6.8		13
1996	7	4.0	0.0	6	114.5	6.5	7	7
1997	9	7.9	2.8	8	117.8	7.3	9	9
1998	16	8.1	2.8	16	125.0	10.0	16	16
1999	15	7.1	2.9	15	117.5	9.7	11	15
2000	21	7.2	2.5	20	116.1	7.1	17	21
2001	10	9.1	4.2	10	120.6	5.5	10	10
2002	17	8.5	4.5	16	121.6	12.0	17	17
2004	20	8.6	2.9	20	122.7	10.4	11	20
2005	5	8.2	4.3	4	113.4	9.7	3	5
2007	12	9.2	3.6	12	116.5	12.0	9	9
Total	168	7.4	3.2	161	118.0	10.4	130	164

**Table 3:** Sample sizes (*n*) of isotopic parameters measured in the blood of adult (≥3 y) female caribou in Denali National Park and Preserve, Alaska.

Year	Isotopic parameters				
	δ^15^N_RBC_ a	δ^15^N_Proteins_ b	δ^15^N_AAs_ c	Δ_RBC-Proteins_ d	Δ_RBC-AAs_ e
1993	14	8	10	8	10
1994	8	1	5	0	4
1995	13	13	13	13	13
1996	7	3	7	3	7
1997	9	5	9	5	9
1998	14	14	16	12	14
1999	15	14	13	14	13
2000	21	18	20	18	20
2001	10	10	7	10	7
2002	16	17	8	16	8
2004	20	20	12	20	12
2005	5	5	5	5	5
2007	3	12	12	3	3

a The isotopic ratios of nitrogen (δ^15^N) in red blood cells.

b δ^15^N in serum proteins.

c δ^15^N in serum amino acids.

d Difference between δ^15^N_RBC_ and δ^15^N_Proteins._

e Difference between δ^15^N_RBC_ and δ^15^N_AAs._

**Figure 5:**
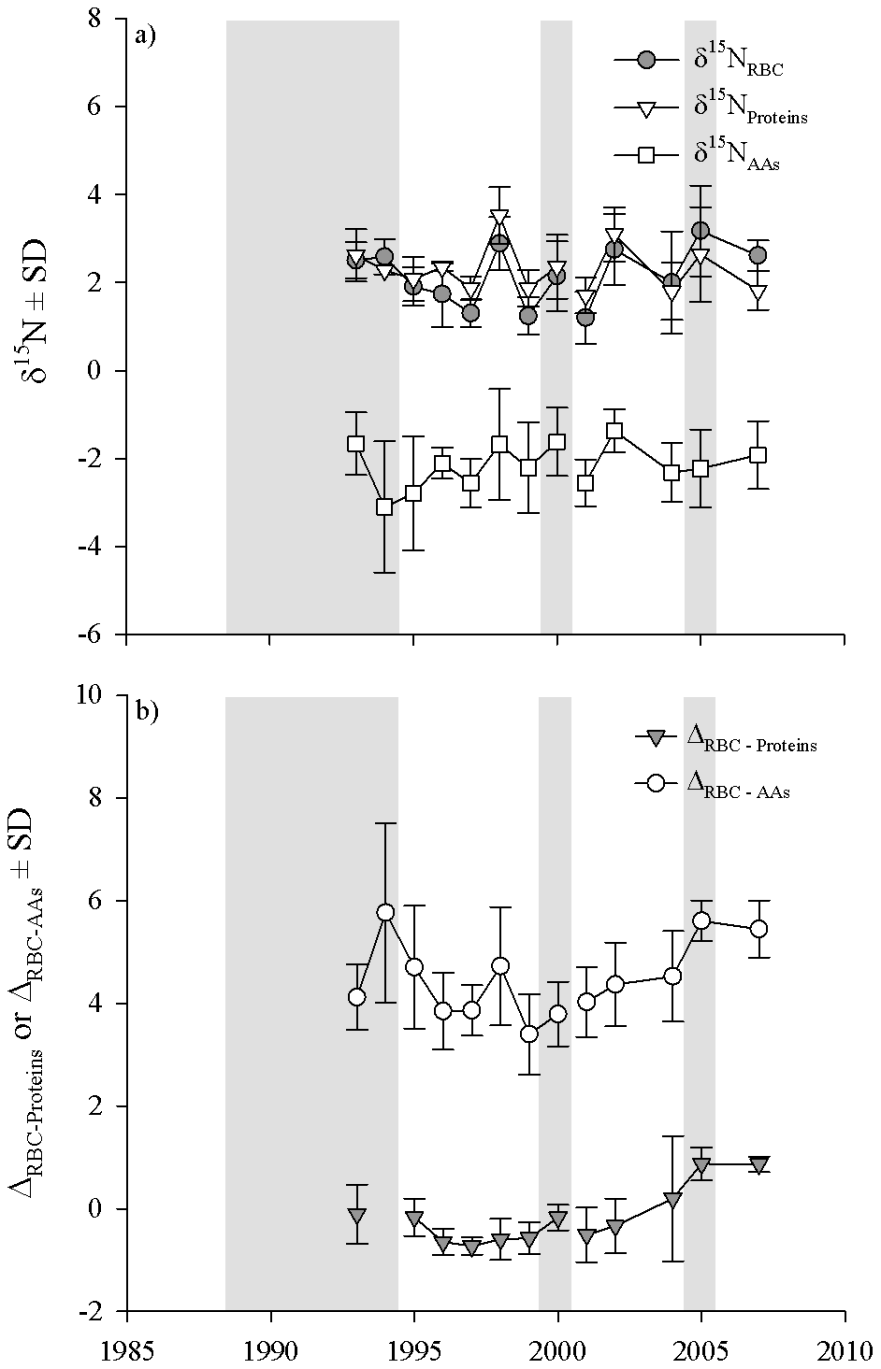
The a) isotopic ratios of nitrogen (δ^15^N in ‰) in the red blood cells (δ^15^N_RBC_), serum proteins (δ^15^N_Proteins_), and serum amino acids (δ^15^N_AAs_) and the b) differences between δ^15^N_RBC_ and δ^15^N_Proteins_ (Δ_RBC-Proteins_) and δ^15^N_RBC_ and δ^15^N_AAs_ (Δ_RBC-AAs_) in adult female caribou from Denali National Park and Preserve, March 1993–2007. Shading denotes winters with above average snowfall.

Isotopic N varied by 4‰ within each fraction of blood across the study period (Fig. 5a, b). The δ^15^N_RBC_ (*n* = 155) averaged 2.1±0.90‰ and ranged from 0.18 to 4.72‰. Similarly, the δ^15^N_Proteins_ (*n* = 140) appeared to track the annual variation in δ^15^N_RBC_ (2.3±0.81‰, range = 0.69 to 4.98‰), while the δ^15^N_AAs_ (*n* = 137) was consistently lower than δ^15^N_RBC_ and δ^15^N_Proteins_ (−2.1±0.99‰, range = −5.97 to 0.58‰). Indeed, these observations at the population level were consistent with comparisons within individual caribou (Fig. 5b) because the Δ_RBC-Proteins_ (*n* = 127) was similar to zero (−0.21±0.71‰, range = −1.51 to 2.41‰) while the Δ_RBC-AAs_ (*n* = 125) was always greater than zero (4.3±1.03‰, range = 1.12 to 7.91‰).

The performance of the regression models appeared to vary with the primary source of N (i.e., body or diet). Models of body protein parameters (δ^15^N_RBC_, δ^15^N_Proteins_, and Δ_RBC-Proteins_) explained ≥62% of the variance (Table 1). Conversely, models that were associated with dietary proteins (δ^15^N_AAs_ and Δ_RBC-AAs_) accounted for substantially less of the variance (≤31%). Year was the most important covariate in the models describing N isotopes of blood fractions, while the effects of age, body mass, and spatial location were significant but much less important (Table 4, 5). Except for δ^15^N_AAs_, year explained the most variation in the other isotopic parameters (all ΔAdj. *R* ^2^<−0.26; Table 4). The effects of year (i.e., βs) on isotopic parameters were not related with September-March snowfall (all *r* ^2^<0.11; all 95% confidence of βs included 0).

**Table 4:** Changes in adjusted *R* ^2^ after each covariate was removed from the global model (Table 1) used to examine the potential sources of variation in isotopic ratios of nitrogen (δ^15^N) for red blood cells (δ^15^N_RBC_), serum proteins (δ^15^N_Proteins_), and serum amino acids (δ^15^N_AAs_), as well as the differences between δ^15^N_RBC_ and δ^15^N_Proteins_ (Δ_RBC-proteins_) and δ^15^N_RBC_ and δ^15^N_AAs_ (Δ_RBC-AAs_) in adult (≥3 y) female caribou, Denali National Park and Preserve, Alaska, March, 1993–2007.

Covariate	δ^15^N_RBC_	δ^15^N_Proteins_	δ^15^Ν_ΑΑσ_	Δ_RBC-proteins_	Δ_RBC-AAs_
Year	−0.46	−0.28	−0.13	−0.42	−0.26
Age+Age^2^	−0.05	0.00	0.00	−0.01	−0.03
Body mass	−0.03	0.00	0.00	0.00	0.00
Winter location	−0.04	−0.03		−0.03	
Late winter location			0.00		0.00
δ^15^N_RBC_		−0.17	−0.15		

**Table 5:** Effects of covariates (β) and their standard errors (SE) on isotopic ratios of nitrogen (δ^15^N) of red blood cells (δ^15^N_RBC_), serum proteins (δ^15^N_Proteins_), and serum amino acids (δ^15^N_AAs_), as well as the differences between δ^15^N_RBC_ and δ^15^N_Proteins_ (Δ_RBC-proteins_) and δ^15^N_RBC_ and δ^15^N_AAs_ (Δ_RBC-AAs_) in adult (≥3 y) female caribou in Denali National Park and Preserve, Alaska, March, 1993–2007; values in bold indicate that confidence intervals (95%) did not include zero.

Covariate		δ^15^N_RBC_		δ^15^N_Proteins_		δ^15^N_AAs_		Δ_RBC-proteins_		Δ_RBC-AAs_	
		β	SE	B	SE	β	SE	β	SE	β	SE
Year^a^	1993	0.16	0.20	**0.80**	0.32	0.56	0.23	−0.33	0.21	−0.42	0.31
	1994	**0.49**	0.22			−**1.16**	0.42			**1.36**	0.43
	1995					−0.46	0.26			0.47	0.27
	1996	−0.10	0.23	0.27	0.23	0.40	0.34	−0.34	0.26	−0.43	0.36
	1997	−**0.95**	0.20	0.15	0.20	0.15	0.33	−**0.57**	0.20	−0.55	0.31
	1998	**0.49**	0.17	**0.66**	0.13	−0.14	0.26	−**0.44**	0.15	0.28	0.27
	1999	−**1.13**	0.17	0.12	0.15	**0.68**	0.28	−**0.52**	0.15	−**1.12**	0.25
	2000	0.15	0.18	−0.01	0.14	0.50	0.25	0.12	0.16	−0.41	0.26
	2001	−**1.25**	0.19	−0.08	0.16	0.21	0.35	−**0.38**	0.15	−0.60	0.34
	2002	**0.34**	0.16	**0.53**	0.12	0.49	0.31	−**0.37**	0.14	−0.29	0.31
	2004	0.31	0.18	−**0.87**	0.14	−0.02	0.26	**1.02**	0.15	−0.12	0.27
	2005	**0.91**	0.32	−0.41	0.25	−0.63	0.43	**0.78**	0.27	**1.07**	0.42
	2007	0.59	0.31	−**0.77**	0.23	−0.58	0.57	**1.03**	0.26	0.76	0.58
Age (y)		−0.18	0.10	−0.14	0.08	0.06	0.15	0.08	0.09	−0.08	0.16
Age^2^ (y)		**0.01**	0.01	0.01	0.01	−0.01	0.01	<−0.01^c^	0.01	0.01	0.01
Body mass (kg)		**0.02**	0.01	<−0.01^c^	0.01	0.01	0.01	0.01	0.01	<−0.01^c^	0.01
Winter location^b^	Easting	−**0.01**	<0.01^c^	−**0.01**	<0.01^c^			<0.01^c^	<0.01^c^		
	Northing	**0.01**	<0.01^c^	0.01	<0.01^c^			<0.01^c^	<0.01^c^		
Late winter^b^	Easting					<−0.01^c^	<0.01^c^			<0.01^c^	0.01
	Northing					<0.01^c^	0.01			0.01	0.01
δ^15^N_RBC_				**0.61**	0.08	**0.61**	0.13				

a Insufficient sample size (*n* = 1) for δ^15^N_Proteins_ in 1994; no winter locations for 1995; and no data for 2003 and 2006.

b Due to large values of coordinates and corresponding small estimates of coefficients, covariates were entered into model as Universal Transverse Mercator coordinate×10^−3^.

c Effects or SEs are too small to be displayed at this level of precision.

The δ^15^N_RBC_ was higher for younger and older animals than prime aged females and increased with body mass (Table 5). The δ^15^N_RBC_ and δ^15^N_Proteins_ were related to spatial location in January as the δ^15^N_RBC_ increased with westerly and northerly directions and δ^15^N_Proteins_ increased with westerly direction (Table 5). The δ^15^N_RBC_ was the second most important covariate for δ^15^N_Proteins_ and the most important covariate in the model for δ^15^N_AAs_ (Table 4) with these measures increasing at similar rates relative to δ^15^N_Proteins_ (Table 5).

## Discussion

We present the first evaluation of a long-term series of factors that influenced δ^15^N within blood fractions, and thereby provide biochemical evidence of the nutrient allocation strategies, as well as physiological and behavioral plasticity, of this northern ungulate. The large inter-annual variances in isotopes and metrics of protein status (Fig. 5) were surprising given the severe winters in the early 1990s (Fig. 4). We clearly overestimated the physiological response of adult females within this low-density population of caribou to a period of historically severe winters. Although we expected isotopic patterns indicative of nutritional restriction for adult females following the severe winters of 1993 and 1994, these biochemical outcomes corresponded well to previous work on these animals [7], [41]. It appears that, even under the harshest conditions, females did not mobilize protein reserves. Therefore, protein restrictions in winter may not be the primary limiting factor for reproduction in this highly vagile ungulate residing at low densities in a predator-rich environment.

The large shifts in δ^15^N of the blood pools among years highlighted important mechanistic factors that shape long-term patterns of isotopic changes. The models and the suite of covariates therein, did quite well in accounting for the variance in pools of N that were likely dominated by body proteins (≥60% of variance explained for δ^15^N_RBC_ and δ^15^N_Proteins_; Table 1). For caribou sampled in March, δ^15^N_RBC_ represented proteins that were deposited or salvaged in late fall and early winter. The evolving picture of δ^15^N_RBC_ within the literature shows that, although varied, estimates generally fall within 1–4‰ with means that are remarkably similar for widely distributed caribou populations. For example, large populations of migratory caribou in Alaska (Western Arctic: 1.5±0.3‰, 32, Central and Western Arctic: 2.2±1.6‰) [54] and Québec (*n* = 60, 2.6±0.5‰) [33] were similar to sedentary, montane populations in central Alaska (this study: 2.1±0.92‰) and the Wrangell-St. Elias Mountains (Chisana: 2.7±0.7‰) [55] as well as north central British Columbia (Pink Mountain: 2.3±0.5‰) [56]. Notwithstanding the similarities among these herds, the substantial inter-annual variance in δ^15^N_RBC_ remains unexplained. We suspect that diet in summer and late fall was strongly influencing δ^15^N_RBC_ as animals stored body protein before winter for the next annual cycle. Thus, space use and diet in late summer-early winter may be useful in resolving some of this variation in δ^15^N_RBC_. Although influential, year remains a covariate that represents a suite of undetermined influences to the isotopic composition of body proteins that integrates N sources over large and varied spatio-temporal scales.

The effects of age, body mass, and location in winter (Table 4) to δ^15^N_RBC_ highlighted some interesting influences to this pool of N. Factors that affect isotopic incorporation rates in blood and tissues [35], such as age and body mass, have been documented in a variety of animals, such as foxes [57], [58], marine mammals [34], birds [59], [60], and fish [60], [61]. Generally, influences of age and body mass to isotopic values are related to individual-level differences in physiological demands, use of habitats, and diets [34], [35]. For females in the Denali herd, the δ^15^N_RBC_ was higher in larger animals as well as younger and older animals (Table 5). It is possible that the effects of age and body mass were correlated. Female caribou experienced a small increase (approx. 11%) in average body mass from 3–5 y and were typically at their average adult body mass by 5 y [7]. The bulk of these blood samples (120 of 171) came from individuals ≥5 y, therefore, for adult females we consider age and body mass effects as unexplained phenomena that deserve future consideration.

The δ^15^N_RBC_ may have been influenced by biogeochemical factors that run along a northwestern gradient across the area. The physiography of the region changes abruptly as the Alaska Range transitions to foothills and lowland habitats toward the northwest (Fig. 3). Corresponding changes in vegetation, geochemistry, and hydrology could influence factors that affect N availability, and thereby δ^15^N, at macro- and micro-scales [30]. Additionally, three species of salmon spawn in the northwestern portion of our study area from early July to November [62]. Marine-derived ^15^N deposited from these salmon may fertilize riparian vegetation, and, thus, enrich the N isotopic profile of caribou forages [63].

The δ^15^N of the serum protein and amino acid pools were strongly influenced by year and, to varying degrees, contributions from body proteins. For both isotopes, interpretations of the year effects were similar to that of δ^15^N_RBC_: year was an important covariate (Table 4) that was not related to snowfall. For δ^15^N_Proteins_, this model performed quite well in accounting for variance within this N pool (78% of variance explained), with most of the additional explained variance (19%) due to the inclusion of δ^15^N_RBC_ as a predictor. The δ^15^N_Proteins_ increased with δ^15^N_RBC_ (Table 5) and covariates that influenced δ^15^N_RBC_ generally shaped δ^15^N_Proteins_ (i.e., winter location). The δ^15^N_Proteins_ closely tracked that of δ^15^N_RBC_ (Fig. 5a), indicating that the pool of serum proteins likely interchanged frequently, and possibly freely, with cellular proteins among serum and red blood cell proteins. Although data on δ^15^N_Proteins_ in wild animals are scant in the literature, serum proteins of reproductive females within migratory caribou herds in Québec were heavier in ^15^N at parturition (*n* = 60, 3.8±0.8‰) [33] than females in late winter from the Denali herd (2.3±0.82‰). This was likely due to the increased recycling of cellular proteins by lactating females in Québec, which is not surprising given the additional protein requirements of lactation and the higher population densities these females experienced throughout the winter and early summer.

Body proteins shaped the patterns of isotopic composition in serum amino acids but this pool of N was likely dominated by dietary proteins. Although year was again a relatively important factor, δ^15^N_RBC_ was the most important predictor of the isotopic composition of the serum amino acid pool (year = 13% of explained variance, δ^15^N_RBC_ = 15%). Yet, this model performed the worst (31% of explained variance) of the model set used to account for variation in δ^15^N of the blood fractions (Table 1). Despite the relative importance of endogenous proteins to this pool, most of unexplained variation in δ^15^N_AA_ was likely due to the large contribution of dietary proteins to this pool. As body proteins turnover, amino acids from these proteins are transported as free amino acids in the serum, thus the pool of serum amino acids comprised a mix of endogenous and exogenous amino acids. The isotopic composition of N in the forage plants of caribou, and thereby exogenous inputs to the serum amino acid pool, was likely quite varied in space and time [54]. Assuming a difference of 2.0±0.6‰ between dietary N and free serum amino acids in caribou (P. S. Barboza, unpublished data), this suggested a winter diet with low δ^15^N values. Indeed, winter diets low in δ^15^N and N that consist primarily of lichens are typical for the Denali herd [54], [64] and caribou in general [65]–[67]. Similar to serum proteins, free amino acids in the serum were also heavier in ^15^N in lactating females in the Québec herds (*n* = 59, −0.4±0.9‰) [33] than in late winter for the Denali herd (*n* = 137, −2.1±0.99‰). With strong implications for protein status, this again suggested that the pool of serum amino acids in Québec females had larger contributions of endogenous proteins than females in the Denali herd, which is not surprising given the conceptual model of isotopic routing (Fig. 1) and aforementioned nutritional stressors.

### Protein status

Despite the variance and severity of winter conditions the caribou in our long-term study endured, we did not detect an isotopic signal of protein limitation in response to winter severity within adult females. To support protein restriction via the conceptual model of isotopic routing (Fig. 1) we expected an increase in Δ_RBC-Proteins_ and a decrease in Δ_RBC-AAs_. However, neither metric of protein status shifted with the severe 1992–93 winter nor were they related to snowfall. On the contrary, in 1993, Δ_RBC-AAs_ was the highest recorded in the 13-year dataset (Table 5, Fig. 5b). In March, forage availability and intake [21] and movement rates [68] may be at or near the annual minima, especially in winters with heavy snowfall. Because these females had not entered the period of steady-state negative N balance we had expected of a wintering ungulate [29] at approximately 8 weeks prior to parturition, these females used dietary proteins to fulfill the demands of tissue maintenance.

The prolonged period of severe winters (Fig. 4a) likely had a limited direct effect on protein status of adult females in this population. Rather, any protein reductions from winter were mediated by topographic diversity, limited intraspecific competition, and reduced lactational costs due to high neonatal losses to predation coupled with a risk-averse strategy of reproductive investment [7], [39], [41]. The spatial diversity of habitats and foraging conditions dampened the influence of winter conditions on this population. Montane environments offer diverse foraging conditions for caribou to attenuate the effects of snow conditions on forage availability. Snowfall, wind, vegetation, and terrain influence foraging conditions, and this spatial heterogeneity in abiotic factors may dampen the effects of density dependence in ungulates [69]. Indeed, coupled with low densities (≤0.3 caribou·km^−2^), the pronounced topographical and vegetative gradients in Denali National Park and Preserve offered ample foraging solutions for caribou. Wintering habitats for caribou included large tracts of lichen-rich spruce forests in the northwest, graminoid-dominated tussock flats in the northeast, as well as the windswept mountains of the northern foothills of the Alaska Range (Fig. 3). Unlike other northern ungulates, such as muskoxen (*O. moschatus*) or Dall sheep (*Ovis dalli*), caribou are well adapted to move amongst these foraging patches through areas of variable snow conditions.

Due to variable winter conditions and high losses of offspring to predation [39], female caribou were risk averse in allocating resources to reproduction [41], [70]. The severe winters of 1989–94 coincided with a sharp decline in population size with pronounced effects to maternal provisioning (Fig. 4c; [7]). Adult females commonly lost their offspring to predation within the first few days of life, but calf losses due to predation continued at older ages throughout the period following the severe winter [39]. As such, most females did not allocate maternal resources towards lactation, while those that had surviving offspring diverted fewer maternal resources towards offspring growth [6]. Thus, adult females did not compromise their protein status in late winter by investing more *in utero* to their offspring. Like other montane ecotypes [55], body masses of adult female caribou in the Denali herd are some of the largest recorded for *Rangifer* spp. Large body mass usually has a positive effect on reproductive success [17], however, at low densities reproductive success is mostly independent of body mass [71]. Prime-aged females maximized reproductive effort in terms of number of pregnancies rather than mass of neonates: Denali caribou produced small offspring relative to their body mass [72]. Indeed, adult female caribou in this population delayed the bulk of reproductive costs until environmental and ecological conditions stabilized [8]. This low investment coming from a parent typically in excellent condition suggests that parents are “protecting” maternal resources. This may have “minimized the maximum detriment” [25] for this montane ungulate by reducing the influence of factors that reduced the number of breeding attempts, which was primarily longevity [73].

### Limitations and implications

Isotope signatures for whole blood, serum, and plasma are typically used to reflect different source materials, but herein we provided the first examination of isotopic routing via pools of blood N (i.e., red blood cells, and serum proteins and amino acids) in wild animals. Blood represents multiple physiological pathways of N, and, thus assessments of bulk fractions may mask informative isotopic differences due to the dynamic nature of isotopic routing. For instance, there appears to be substantial variance in the isotopic sources within C and N of essential and non-essential amino acids [74]–[76]. This work may have benefited by isolated fractions of both C and N in the serum amino acid pool into their individual amino acid components, but due to the conversion and production of all essential amino acids via bacterial metabolism in the rumen, it remains to be seen whether this approach will be fruitful for ruminants. However, if a specific amino acid(s) was identified that limits bacterial metabolism (e.g., leucine or methionine) [31], then there may be compound-specific approaches to trace exogenous and endogenous contributions to the pool of free amino acids in the blood of ruminants.

The substantial inter- and intra-annual variation in δ^15^N observed in our study could have been the effect of physiological and ecological variations among individuals on the metabolic routing and consequent relative contributions of novel and endogenous resources to N in the non-essential amino acids in caribou red blood cells. Previous studies [77]–[79] have noted the influence of reproductive status, body condition, and dietary composition on the metabolic routing of dietary macromolecules in a variety of animal species. Like dietary sources, it is possible that physiological and ecological conditions influence the routing of macromolecules from endogenous sources and the consequent mixture of endogenous and novel resources to the non-essential amino acids in caribou tissues. If these processes occurred in Denali caribou, we would expect divergences in physiological conditions among individuals to cause inter-annual variations in δ^15^N_RBC_ (and potentially δ^15^N_AA_). Because caribou are a gregarious and generalist species, we would expect variations in δ^15^N_RBC_ caused by contrasts in dietary inputs to operate on the population scale, thus, resulting in intra-annual differences in δ^15^N_RBC._ The isotopic model presented herein is conceptual, and by nature, deserves more rigorous evaluations. It is possible that our conceptual model is not sensitive enough to identify small but physiological significant shifts in protein status. Forthcoming work from captive research, however, suggests that this is unlikely and will highlight further insights gained from this model of isotopic routing in northern ungulates. Accordingly, we recommend that future research to examine the sources of inter- and intra-annual variance in the isotopic ecology of large herbivores should consider the conceptual model of isotopic routing presented herein, compound specific assessments, or effects of individual physiology and dietary ecology on metabolic routing.

This biochemical evidence supports previous work on this population while continuing to enhance our understanding of some of the environmental, ecological, and physiological mechanisms that affect population dynamics within montane caribou. Year, maternal (age and body mass), and spatial factors (winter location) shaped patterns of variance in δ^15^N within this montane ungulate, while demonstrating little isotopic evidence of reliance on maternal proteins even during severe winters. Caribou continue to demonstrate considerable physiological and behavioral plasticity that undoubtedly contributes to the resilience of this population in the presence of often harsh and varied environmental and ecological conditions that are common of multi-predator multi-prey systems in northern and montane environments.
